# Potential Effects of Lifelong Team Handball and Football Training and Nutritional Habits on Bone Health and Body Composition in Elderly Women

**DOI:** 10.3390/jfmk9030159

**Published:** 2024-09-07

**Authors:** Domenico Martone, Jeppe Foged Vigh-Larsen, Daniela Vitucci, Malte Nejst Larsen, Morten Bredsgaard Randers, Jens Lykkegaard Olesen, Magni Mohr, Annamaria Mancini, Peter Krustrup, Pasqualina Buono

**Affiliations:** 1 Department of Economics, Law, Cybersecurity and Sports Sciences, University Parthenope, 80035 Naples, Italy; 2 CEINGE-Biotecnologie Avanzate Franco Salvatore S.c.a.r.l, 80131 Naples, Italy; daniela.vitucci@assegnista.uniparthenope.it (D.V.); annamaria.mancini@uniparthenope.it (A.M.); 3 Department of Sports Science and Clinical Biomechanics, SDU Sport and Health Sciences Cluster (SHSC), University of Southern Denmark, 5230 Odense, Denmark; jeppefoged.vighlarsen@ugent.be (J.F.V.-L.);mnlarsen@health.sdu.dk (M.N.L.); mranders@health.sdu.dk (M.B.R.); mmohr@health.sdu.dk (M.M.); pkrustrup@health.sdu.dk (P.K.); 4 Department of Medical, Human Movement and Well-Being Sciences, University Parthenope, 80133 Naples, Italy; 5 Center for General Practice, Aalborg University, Fyrkildevej 7, 9220 Aalborg, Denmark; jlo@dcm.aau.dk; 6 Centre of Health Sciences, Faculty of Health, University of Faroe Islands, FO-100 Tórshavn, Faroe Islands

**Keywords:** lifelong training, elderly, female, bone health, team handball, international football, soccer

## Abstract

Background/Objectives: The aim of this study was to evaluate the effects of lifelong team handball/football training on regional bone health and body composition in elderly women. Methods: Seventeen elderly women team handball/football players (65.9 ± 5.7 years) and twenty-one untrained age-matched women (controls) (67.7 ± 5.1 years) participated. Whole-body and regional dual-energy X-ray absorptiometry scans of arms, legs, and lower spine (L1–L4) were performed. Results: We observed 8% and 9% higher bone mineral density (BMD) and bone mineral content (BMC), respectively, at the whole-body level and in the legs and 11.5% higher BMC in the legs in team handball/football players compared to untrained age-matched controls (*p* < 0.05). Higher total and leg lean body mass (*p* < 0.05), along with lower total body fat percentage *(p* < 0.05) and higher T- and Z-scores, markers of fragility risk fracture (0.294 ± 1.461 vs. −0.538 ± 1.031; 1.447 ± 1.278 vs. 0.724 ± 0.823, respectively), were also found in team handball/football players compared to controls (*p* < 0.05). No significant differences in nutritional habits were observed between groups. Conclusions: Our study suggest that the beneficial effects of lifetime handball/football practice on bone preservation in elderly women occur independently from nutritional intake, which emphasize the potential role of team sports in osteoporosis prevention. Future studies should focus on the cofounding factors and causative mechanisms mediated by team sport practice in osteoporosis prevention.

## 1. Introduction

Aging is a physiological process characterized by a progressive decline in biological functions which are often enhanced by other conditions such as sedentary lifestyle and an inadequate nutritional regime [1,2,3,4,5]. These processes can determine a loss of muscle and bone mass and the onset of metabolic, cardiovascular and neurodegenerative diseases [6,7,8,9]. In particular, osteoporosis is one of the most common metabolic disorders in women aged >50 years when entering the postmenopausal period with potential consequences for overall health and quality of life due to increased risks of falls and bone fractures [10,11]. In 2019, in the European Union alone, 25.5 million women and 6.5 million men were estimated to have osteoporosis; 4.3 million new fragility fractures were sustained comprising mainly hip, vertebral and forearm fractures [12]. Moreover, it has been estimated that more than 50% of patients affected by bone fractures never completely regain their previous functional status, and about 20% of them meet premature death [9,12].

Physical activity can be particularly beneficial for the gaining and maintenance of healthy strong bones especially if started at an early age [13]. Regular exercise has shown to be effective in maximizing peak bone mass in girls and minimizing bone loss in postmenopausal women [14]. Regarding the type of exercise, it has been shown that repeated high-impact, resistive exercises such as jumping, aerobics, running and strength training (e.g., weightlifting) have positive effects on bones in all age groups [15]. Football is a sport that repeatedly alternates accelerations and decelerations, changes of direction, jumps and sprints that generate an osteogenic effect through traction and reaction forces resulting from muscle contractions and mechanic impact on the ground [16]. Indeed, it has been shown that women aged 18–65 years that regularly exercised in recreational football training for 4–12 months improved bone mineralization by 1–5% and 1–2% in lower limb and whole-body, respectively [17]. Similar results have been observed in professional female football players compared to untrained women [18]. Team handball, which also combines aerobic and anaerobic training, has recently been proposed as an activity favoring bone health [19,20,21,22]. One study found that 16 weeks of recreational handball training was effective to stimulate bone turnover as well as postural balance in postmenopausal women without previous sports experience [19]. Another study observed an increase by 0.8% in proximal femur body mineral density (BMD) after 12 weeks of regular participation in small-sided team handball in young trained compared to untrained women [20]. Higher BMD values in the lumbar spine, lower extremity, and right upper extremity were also detected in children and adolescents involved in team handball compared to controls [21,22].

Although the benefits of physical activity in the prevention and treatment of non-communicable diseases (NCDs) are well established, the average worldwide prevalence of inactivity is still high in women (27%) and men (20%), and this trend is more pronounced in the elderly [23,24]. Given the importance of physical activity for bone health from an early age, it is useful to promote strategies for long-term adherence of sport participation, especially for females. Indeed, we recently observed that lifelong male football players had in some body regions higher BMD and bone mineral content (BMC) compared to age-matched untrained controls [25]. Similar results were also reported by Hagman et al. [26]. Studies evaluating the effects of lifelong female’s sport participation on bone health are scarce, and until now only Hagman et al. [27] analyzed the effects of lifelong handball training in elderly female players reporting a higher BMD in whole-body, leg and lumbar spine than age-matched untrained controls [27].

However, not only physical activity but also other lifestyle and environmental factors, as well as diet, may play an important role in bone health [28]. Mainly in older people, vitamin D, calcium, and magnesium supplements are used in order to limit the age-related bone density loss [29,30]. No previous studies involving lifelong female football and handball players have evaluated their dietary habits.

Thus, the first aim of the present controlled cross-sectional study was to evaluate the effects of lifelong football and handball training on BMD and BMC in the whole-body and in different regions, including arms, legs and lower spine, in a group of elderly handball/football players (VHF) in comparison with age-matched untrained females (CG). The secondary aim was to analyze the daily caloric and nutrient intake associated with the sport training in VHF compared to CG.

## 2. Materials and Methods

### 2.1. Participants

All participants were women in post-menopausal age. VHF were recruited through The Danish Football Association (Dansk Boldspil-Union; DBU) and The Danish Handball Federation (Dansk Håndbold), with help from coaches in local football and handball clubs near Odense, Denmark. CG women were recruited by advertisements in social media and local newspapers near Odense, Denmark; they had not participated in regular physical activity and sports during their life prior to the recruitment, as assessed by an interview.

The inclusion criteria for VHF women included regular handball and/or football training for the last 12 months, as well as for a minimum of 10 years in total in their lifetime. Exclusion criteria were unbalanced diets such as vegan, vegetarian, ketogenic ergogenic, vitamin supplementation (except vitamin D and calcium intake within the recommended dosage for their age) and specific drugs (e.g., antibiotics) and hormone supplements (e.g., corticosteroids). Moreover, no participants had chronic diseases, although a mild hypertension or hyperlipidemia were not exclusion criteria. All subjects provided written informed consent before participation. The study was conducted in accordance with the guidelines of the Declaration of Helsinki and approved by the regional ethics committee for Southern Denmark (ID S-20180165 approved on 28 June 2019).

### 2.2. Testing Procedures

The participants were invited to the laboratory on a single occasion in the morning between 7 a.m. and 9 a.m. after an overnight fast, and for VHF, without having trained in the last 48 h. The testing procedures included in succession: the anthropometric measurements were followed by filling out a food habits questionnaire and dual-energy X-ray absorptiometry (DXA). All testing procedures were carried out by medical doctors and sports scientists.

#### 2.2.1. Anthropometric Measurements and Food Habits

All participants were assessed for body weight and height. The measurements were recorded with subjects in bare feet and wearing light clothing, using standardized equipment (InBody 230 analyzer, Seoul, Republic of Korea) to the nearest 0.1 kg for body weight and 0.1 cm for height. Body Mass Index (BMI) was calculated as body weight in kilograms divided by the square of height in meters (kg/m^2^).

To estimate the daily food intake, participants completed the 3-day food diary for 2 weekdays and 1 weekend day (e.g., Monday, Thursday, and Saturday), reporting the time of consumption and the type and amount of food eaten. The data were processed using Winfood software version 3.14.3 (Medimatica S.u.r.l., Colonnella, Italy).

#### 2.2.2. BMD, BMC and Body Composition Evaluation

All measurements were conducted by DXA scan in accordance with standard procedures (Prodigy Advance, Lunar Corporation, Madison, WI, USA). BMD (g/cm^2^) and BMC (g) were evaluated in both arms and legs, whole-body and lower spine (L1, L2, L3 and L4), along with whole-body T- and Z-scores. Total lean body mass (kg), total body fat mass (kg), total body fat percentage (%), leg lean mass (kg), android body fat (%) and gynoid body fat (%) were also assessed using DXA scanning. This procedure has been standardized as described in [31]. Briefly, subjects were asked to empty their bladder and to remove metal objects to minimize interference with the results. All analyses were performed using enCORE software version 15 (Lunar Corporation, Madison, WI, USA). The effective radiation dose for scans was 10.84 mSv in total.

### 2.3. Statistical Analyses

Using G*power 3.1 software [32], we determined the number of participants required per group to have a statistical power of 0.80 or higher with an effect size of 0.5 at thesignificance level alpha = 0.05. Considering the results reported by Hagman et al. [27], we calculated a simple size of 15 women for VHF and 15 women for CG to detect a 2% in BMD and BMC due to the osteogenic effect induced by football and handball training. Comparisons between groups were determined with Student’s *t*-test for all normally distributed variables or with the Mann–Whitney test for non-parametric variables. Effect size (ES) was also calculated and interpreted as suggested by Hopkins et al. [33]: <0.2 trivial; >0.2 and <0.6 small; >0.6 and <1.2 moderate; >1.2 and <2.0 large and >2.0 very large. All data are expressed as mean standard deviation (SD). All values are reported as mean ± standard deviation. The level of significance was set at *p* < 0.05, and all analyses were performed using Jamovi software (version 2.3.28).

## 3. Results

According to inclusion and exclusion criteria, a total of 40 women were recruited; two of them declined for personal reasons. Thirty-eight women took part in the study, with seventeen belonging to the VHF group (65.9 ± 5.7 y) and twenty-one to the CG group (67.7 ± 5.1 y). In particular, n = 7 played handball and n = 10 football regularly (≥1 session per week) for more than 30 years (35.3 ± 17.5 y; range: 10–59 y).

### Whole-Body and Regional BMD and BMC

Whole-body and regional BMD and BMC assessments are reported in Table 1 and Table 2, respectively.

Whole-body and legs BMD were 8% and 9% higher in the VHF compared to the CG (*p* < 0.048, ES = 0.666; *p* = 0.043 ES = 0.684), respectively. T- and Z-scores were higher in VHF compared to CG (0.294 ± 1.461 vs. −0.538 ± 1.031; *p* = 0.047 and 1.447 ± 1.278 vs. 0.724 ± 0.823; *p* = 0.042, respectively; Table 1). Furthermore, the VHF showed 11.5% higher legs BMC compared to the CG (*p* = 0.033 and ES = 0.725; Table 2). No significant differences in age, height, total body mass and BMI between the VHF and the CG were found.

VHF showed higher total lean body mass (2.9 kg; *p* = 0.035; ES = 0.714), higher leg lean mass (1.3 kg; *p* = 0.019; ES = 0.804) and 4.9% lower total body fat percentage (*p* = 0.047; ES = 0.671), together with 5% lower gynoid body fat percentage (*p* = 0.020 and ES = 0.830) compared to the CG (Table 3).

The 3-day food diary was completed by 11 members of the VHF and 14 of the CG. All of them were taking calcium and vitamin D supplements within the recommended doses for their age. There were no significant differences (*p* > 0.05) between groups in daily caloric and nutrient intake (Table 4).

## 4. Discussion

The present study aimed to evaluate the effects of lifelong handball and football training on BMC and BMD in whole-body and in different body regions, including arms, legs and the lower spine, in a group of VHF compared to untrained age-matched females, and as a secondary aim, to analyze the effect of caloric intake and diet composition in association to lifelong training on bone health. VHF with regular participation in handball/football training had 9% and 11.5% higher BMD and BMC values in legs and 8% higher BMD values in whole-body BMD compared to the CG. Moreover, T- and Z-score values were higher in VHF compared to CG. We also found a leaner body composition, with higher total and legs lean body mass and a lower percentage of total and gynoid body fat in VHF compared to the CG.

Bone modeling due to physical activity obtained by a mechanical load should be multi-directional, intermittent, high in magnitude and rapidly applied. The magnitude of bone adaptations through training depends on type, intensity, frequency and duration. In fact, it has been demonstrated that athletes involved in high-impact sports (e.g., basketball, volleyball, gymnastics, etc.) had higher BMD values compared to low- or no-impact sports training (e.g., swimming, cycling, yoga, etc.) [34].

The regular participation in handball/football training for more than 30 years could explain the osteogenic effect observed in the VHF compared to the CG. Our results are in line with Hagman et al., who reported that lifelong handball female players aged 60–80 years had 8% and 5% higher mean legs and whole-body BMD, respectively, and 13% higher mean legs BMC compared to age-matched untrained subjects [27]. In contrast, we did not find a significant difference in lower spine BMD and BMC between VHF and CG. In particular, lower spine BMD and BMC values reported by Hagman et al. resulted higher than those observed in our sample, although all participants live in Denmark. It has been observed that menopausal early onset and duration negatively affects bone density in different regional sites [35,36]. Thus, we can speculate that the discrepancy in lower spine BMD and BMC values between our and Hagman et al.’s results may be ascribed to the cofounding factors of onset and duration of menopause.

In other similar studies [25,26] involving men aged 60–85 years, it was also observed that lifelong football players had higher BMD and BMC ranging from 6.3% to 33.2% in whole-body, arms, legs, proximal femur and lower spine compared to the CG.

A positive effect on bone health was also reported in senior athletes engaged in the 2005 National Senior Games (the Senior Olympics). In particular, Leigey et al. [37] observed that senior athletes participating in high-impact sports such as basketball, volleyball, and triathlons, showed higher BMD T-score value (averaged 0.4 ± 1.3) compared with those involved in low-impact sports such as cycling, swimming, and bowling (averaged −0.1 ± 1.4).

Besides better values of BMD and BMC, VHF also showed improved body composition (2.9 and 1.3 kg higher total lean body mass and leg lean mass) compared to CG. Although genetics play a role in regulating bone mass and lean body mass, lifelong training in handball or football could also contribute. Moreover, the natural selection process could influence the selection of women with greater leg lean mass to participate in sports like handball and football, contributing to the results observed in the VHF group. This is in agreement with a recent cross-sectional study evidencing higher BMD and BMC values as a better body composition in lifelong male football players compared to CG [25]. In this study, it was pointed out that during a football match, more than half of the time session (70%) is performed at a heart rate (HR) range of 76–89% of individual theoretical maximum HR, with a mean of 419 and 428 accelerations and decelerations, respectively [25]. Thus, an increase in bone mineralization and lean body mass could be ascribed to football demands characterized by various intense actions such as turns, jumps and sprints in different directions, which determine high-impact force on the bones [17,20,38].

Handball and football are team sports characterized by intermittent activities and require continuous changes of direction, with handball characterized by more jumping compared to football [17,20,39]. It has been observed that during football training, the mean and peak heart rates correspond to 79 and 96% of the maximum heart rate (HRmax), respectively, in elderly women [40]. Thus, it can be hypothesized that, similar to football, handball training also requires intense action in elderly women that in turn induces bone stimulation and improves bone health.

The expression of sclerostin, osteocalcin, P1NP and CTX-1, and bone turnover markers (BTMs) [41] was affected by the type, intensity and amount of regular exercise [42]. In particular, it was observed that lifelong handball female players had 14% lower expression levels of circulating sclerostin, a negative regulator of both bone mass and bone strength, than age-matched untrained females [27]. In addition, muscle contraction induces per se the release of myokines and specific enantiomers, as L-BAIBA, that independent from the mechanical loads and ascribed to the specific exercise, prevents osteocytes senescence and counteracts age-related bone loss [43,44,45]. We can speculate that the regular engagement in high-impact sports such as handball/football over a lifetime (>30 years) may have contributed to the improvement of BMD and BMC, activating both mechanical and osteogenic pathways, thus preventing osteogenic senescence in VHF compared to the CG.

In cross-sectional studies, it is not clear if the improvement in BMD and BMC values is to be ascribed only to regular handball/football training or to other confounding factors, such as nutritional habits. Here, we evaluated the food habits in the participants. For instance, it is well known that the intake of vitamin D and calcium is helpful in promoting the bone formation/remodeling process, mostly in elderly people [46,47]. Since both groups showed no significant differences in food habits, higher values of BMD and BMC were observed in the VHF compared to the CG, reinforcing the role of handball/football training in preserving bone health over a lifetime. Moreover, we observed that both groups had an unbalanced nutrient intake in fats which, besides being associated with cardiometabolic diseases, may result in decreased BMD and is associated with an elevated risk of fractures in postmenopausal women [48]. Interestingly, higher BMD and BMC values observed in VHF compared to CG underlines the positive effects of the regular practice of handball/football exercise on bone loss, counteracting the effects of a diet rich in fat.

Osteoporosis is one of the major public health problems worldwide, mostly in postmenopausal women who experience a larger bone loss in old age than men [11]. Specific exercise guidelines aimed to improve bone mass and reduce the risk of fractures have failed to achieve the goal due to low population compliance [49]. It is important to maximize bone mass and bone strength at an early age to reduce the risk of osteoporosis in later life and counteract age-related bone loss. In the present study, in addition to higher BMD and BMC and a better lean body mass, VHF showed higher T- and Z-scores compared to CG. Therefore, it can be assumed that handball and football training represent not only a powerful osteogenic stimulus, in the prevention of osteoporosis and fracture risks, but also promote better postural control with reduction of the risk of falling in elderly women [19,27,50]. Moreover, since handball and football are team sports, the social and enjoyable factor of the game may enhance participation and motivation to continue.

Confounding factors such as the variability in genetics, sex hormone levels and modifiable factors (e.g., alcohol assumption or smoking) affect the BMD and BMC. Heritable factors could explain about 60–80% of the variability in bone mass and osteoporosis risks while the remaining 20% depend on environmental factors and sex hormone levels during puberty [51,52]. Further, several studies have shown that the pubertal timing affects peak bone mass, with greater BMD and BMC values associated with earlier age of pubertal onset in girls [28]. Furthermore, growing evidence supports the positive role of physical activity levels, especially during the late childhood and peripubertal years, for the growth and maintenance of bone mass over time [28,53]. Alcohol consumption (over one glass per day in women or over two glasses per day in men) and smoking behaviors were associated with a decrease in strength and bone mass during aging, if these behavior are early and continued into adulthood [49].

In the present study, we did not look at the cofounding factors, and this is a limitation. Thus, it will be interesting in the future to investigate the influences of lifestyle factors (e.g., smoking habits, physical activity in daily life, in addition to sports training) together with pubertal timing, hormonal and molecular mechanisms induced by different team sports practice in order to increase knowledge on the causative effects of different team sports on bone health.

## 5. Conclusions

The results of the present study showed that lifelong football and handball training (>30 years) have a potential effect on improving body composition and bone health compared to age-matched untrained elderly women. Interestingly, the improvement in body composition and in BMC and BMD were not ascribed to different food habits or supplementation in elderly women participating in the study. Our results underline the potential role of team handball and football training independent of nutritional habits in the reduction of osteoporosis and fracture risks and the promotion of healthy aging in elderly women. Future studies will analyze the effects of team sports on confounding factors associated with BMD and BMC and osteoporosis prevention and provide data on causative mechanisms.

## Figures and Tables

**Table 1:** Bone mineral density (g/cm^2^) in the arms, legs, whole-body and lower spine in lifelong female handball and football players (VHF) and the age-matched untrained control (CG) participating in the study (mean ± SD).

Variable (Units)	VHF (n = 17)	CG (n = 21)	t-Value	*p*-Value	ES	95% CI	
						Lower	Upper
Arms	0.830 ± 0.109	0.782 ± 0.073	0.387	0.124	0.514	−0.156	1.170
Legs	1.108 ± 0.148	1.016 ± 0.122	0.589	0.043	0.684	−0.006	1.356
Whole-body	1.109 ± 0.147	1.026 ± 0.103	0.013	0.048	0.666	−0.022	1.336
L1	0.950 ± 0.166	0.948 ± 0.120	−0.272	0.959	0.016	−0.622	0.656
L2	1.052 ± 0.213	1.005 ± 0.159	−0.699	0.438	0.255	−0.393	0.897
L3	1.448 ± 0.216	1.086 ± 0.126	−0.919	0.302	0.341	−0.313	0.986
L4	1.649 ± 0.254	1.096 ± 0.170	−0.341	0.328	0.323	−0.330	0.962
L1–L4	1.089 ± 0.203	1.040 ± 0.132	0.891	0.379	0.291	−0.361	0.934
T-score	0.294 ± 1.461	−0.538 ± 1.031	2.055	0.047	0.670	−0.018	1.341
Z-score	1.447 ± 1.278	0.724 ± 0.823	2.111	0.042	0.688	−0.002	1.361

Abbreviations: L1 to L4, lumbar spine vertebrae; ES, effect size; CI, confidence interval of ES.

**Table 2:** Bone mineral content (g) in the arms, legs, whole-body and lower spine in lifelong female handball and football players (VHF) and the age-matched untrained control (CG) participating in the study (mean ± SD).

Variable (Units)	VHF (n = 17)	CG (n = 21)	t-Value	*p*-Value	ES	95% CI	
						Lower	Upper
Arms	272.8 ± 47.2	246.9 ± 39.7	−1.238	0.075	0.597	−0.082	1.261
Legs	831.1 ± 120.1	744.9 ± 117.8	0.414	0.033	0.725	0.029	1.402
Whole-body	2205.9 ± 325.9	2026.8 ± 260.3	−0.586	0.068	0.614	−0.067	1.280
L1	11.4 ± 2.6	11.2 ± 2.1	−0.346	0.734	0.111	−0.530	0.750
L2	13.0 ± 3.2	12.8 ± 2.3	−1.258	0.776	0.093	−0.548	0.732
L3	16.3 ± 4.9	15.1 ± 2.2	−1.539	0.346	0.311	−0.341	0.955
L4	18.9 ± 6.5	17.3 ± 3.1	−0.979	0.424	0.264	−0.386	0.906
L1–L4	56.7 ± 15.6	56.4 ± 8.8	0.809	0.328	0.323	−0.330	0.968

Abbreviations: L1 to L4, lumbar spine vertebrae; ES, effect size; CI, confidence interval of ES.

**Table 3:** Anthropometry and body composition in lifelong female team handball and football players (VHF) and age-matched untrained controls (CG) participating in the study (mean ± SD).

Variable (Units)	VHF (n = 17)	CG (n = 21)	t-Value	*p*-Value	ES	95% CI	
						Lower	Upper
Age (years)	65.9 ± 5.7	67.7 ± 5.0	−3.657	0.313	0.334	−0.321	0.978
Height (cm)	164.3 ± 5.0	165.0 ± 7.4	−0.275	0.324	0.108	−0.747	0.534
Total body mass (kg)	66.3 ± 10.8	66.5 ± 9.9	−0.546	0.958	0.172	−0.656	0.622
BMI (kg/m^2^)	24.5 ± 3.3	24.4 ± 3.4	−0.484	0.969	0.013	−0.627	0.652
Total lean body mass (kg)	42.0 ± 4.3	39.1 ± 4.1	0.710	0.035	0.714	0.019	1.390
Legs lean mass (kg)	14.3 ± 1.6	13.0 ± 1.7	0.825	0.019	0.804	0.098	1.491
Total body fat (%)	33.2 ± 7.6	38.1 ± 7.0	−1.550	0.047	0.671	0.018	1.341
Total body fat mass (kg)	21.8 ± 7.8	24.8 ± 7.9	−1.063	0.241	0.389	−0.270	1.036
Android body fat (%)	34.5 ± 12.1	41.3 ± 10.9	−1.267	0.080	0.588	−0.091	1.250
Gynoid body fat (%)	36.9 ± 6.2	41.9 ± 5.9	−1.451	0.020	0.830	0.120	1.520

Abbreviations: L1 to L4, lumbar spine vertebrae; ES, effect size; CI, confidence interval of ES.

**Table 4:** Daily caloric and nutrient intake of lifelong female team handball and football players (VHF) and the age-matched untrained control (CG) participating in the study (mean ± SD).

Variable (Units)	VHF (n = 11)	CG (n = 14)	*p*-Value
Carbohydrates (%)	35.1 ± 6.1	34.7 ± 6.1	0.892
Lipid (%)	46.7 ± 5.5	47.1 ± 5.5	0.867
Protein (%)	18.4 ± 2.2	18.6 ± 2.5	0.849
Vitamin D (mcg)	2.2 ± 1.1	2.5 ± 1.7	0.558
Calcium (mg)	786 ± 241	724 ± 215	0.536
Alcohol (g)	4.7 ± 6.8	10.0 ± 10.1	0.198
Caloric intake (kcal/d)	1647 ± 207	1807 ± 388	0.258

## Data Availability

Data generated or analyzed during this study are available from the corresponding author upon reasonable request.
